# CNS tumor stroma transcriptomics identify perivascular fibroblasts as predictors of immunotherapy resistance in glioblastoma patients

**DOI:** 10.1038/s41525-023-00381-w

**Published:** 2023-10-26

**Authors:** Maksym Zarodniuk, Alexander Steele, Xin Lu, Jun Li, Meenal Datta

**Affiliations:** 1 Department of Aerospace and Mechanical Engineering, University of Notre Dame, Notre Dame, IN USA; 2 Department of Electrical Engineering, University of Notre Dame, Notre Dame, IN USA; 3 Department of Biological Sciences, University of Notre Dame, Notre Dame, IN USA; 4 Department of Applied and Computational Mathematics and Statistics, University of Notre Dame, Notre Dame, IN USA

**Keywords:** CNS cancer, CNS cancer

## Abstract

Excessive deposition of extracellular matrix (ECM) is a hallmark of solid tumors; however, it remains poorly understood which cellular and molecular components contribute to the formation of ECM stroma in central nervous system (CNS) tumors. Here, we undertake a pan-CNS analysis of retrospective gene expression datasets to characterize inter- and intra-tumoral heterogeneity of ECM remodeling signatures in both adult and pediatric CNS disease. We find that CNS lesions – glioblastoma in particular – can be divided into two ECM-based subtypes (ECM^hi^ and ECM^lo^) that are influenced by the presence of perivascular stromal cells resembling cancer-associated fibroblasts (CAFs). Ligand-receptor network analysis predicts that perivascular fibroblasts activate signaling pathways responsible for recruitment of tumor-associated macrophages and promotion of cancer stemness. Our analysis reveals that perivascular fibroblasts are correlated with unfavorable response to immune checkpoint blockade in glioblastoma and poor patient survival across a subset of CNS tumors. We provide insights into new stroma-driven mechanisms underlying immune evasion and immunotherapy resistance in CNS tumors like glioblastoma, and discuss how targeting these perivascular fibroblasts may prove an effective approach to improving treatment response and patient survival in a variety of CNS tumors.

## Introduction

CNS tumors comprise a highly heterogeneous group of malignancies that originate from different cell types and affect various anatomical structures. Despite recent advances in immunotherapeutic approaches to treat solid tumors, survival for many types of CNS cancers has not improved in the past 10 years^1,2^. Thus, there is an urgent need for a better understanding of the underlying mechanisms governing disease progression and treatment resistance.

Tumor stroma, composed of extracellular matrix (ECM) and specialized connective tissue cells that include fibroblasts, has been shown to shape antitumor immunity and response to immunotherapy across a broad range of epithelial tumors such as breast, colon, and pancreatic carcinomas^3–7^. However, little is known about the immunomodulatory roles of the brain tumor stroma, and the distinct cellular makeup of the brain makes it challenging to extrapolate findings from cancers arising in peripheral organs.

Parenchyma of the CNS is often regarded as “fibro-privileged” since fibrogenic cells are absent in the normal neural parenchyma and are instead restricted to perivascular and meningeal niches^8,9^. This is in line with the lack of fibrillar collagens in the brain tumor parenchyma^10^. It has been shown in a number of neuropathologic conditions such as traumatic brain injury, ischemic stroke, and multiple sclerosis that perivascular stromal cells can detach from the vasculature, migrate into the parenchyma, and form a dense fibrotic scar^11^, thereby inhibiting axonal regeneration^12^. However, the exact contribution of perivascular stromal cells to the brain tumor ECM remains unclear. This is partly due to the limited evidence supporting the existence of fibroblasts in primary brain tumors that are otherwise commonly found in the ECM-rich stroma of highly desmoplastic extracranial tumors such as breast, prostate, and pancreatic carcinomas^13–15^. Both extracranial cancer-associated fibroblasts (CAFs) and CAF-derived ECM have been linked to suppression of antitumor immune responses^16–18^; however, evidence supporting their immunosuppressive roles in brain tumors is lacking. Therefore, a better understanding of the cellular origin and role of stroma in the brain tumor microenvironment (TME) can reveal novel mechanisms of immune escape and guide the design of new strategies to improve immunotherapeutic outcomes in brain tumors.

To this end, we performed a meta-analysis of publicly available gene expression datasets from multiple cohorts of pediatric and adult primary CNS tumors. We focused our attention on “core matrisome” – the ensemble of genes encoding structural components of the ECM, such as collagens and proteoglycans^19^, the latter of which constitute a major component of brain tumor ECM^10,20^. Through unsupervised analysis of gene expression data, we identified a subset of glioblastoma (GBM) tumors characterized by overexpression of transcripts of extracellular matrix (ECM) components and the presence of perivascular stromal cells resembling CAFs. This ECM-high signature was conserved across a broad range of CNS malignancies and predicted poor patient survival in a subset of tumors. Interrogation of retrospective data from recent anti-PD1 immunotherapy trials^21,22^ revealed an association between the presence of perivascular fibroblasts and immunotherapy resistance. In this study, we propose several mechanisms by which perivascular fibroblasts can contribute to immune evasion and immunotherapy resistance in CNS disease.

## Results

### Unsupervised analysis of extracellular matrix expression in glioblastoma reveals two states that are conserved in other CNS tumors

**Fig. 1:**
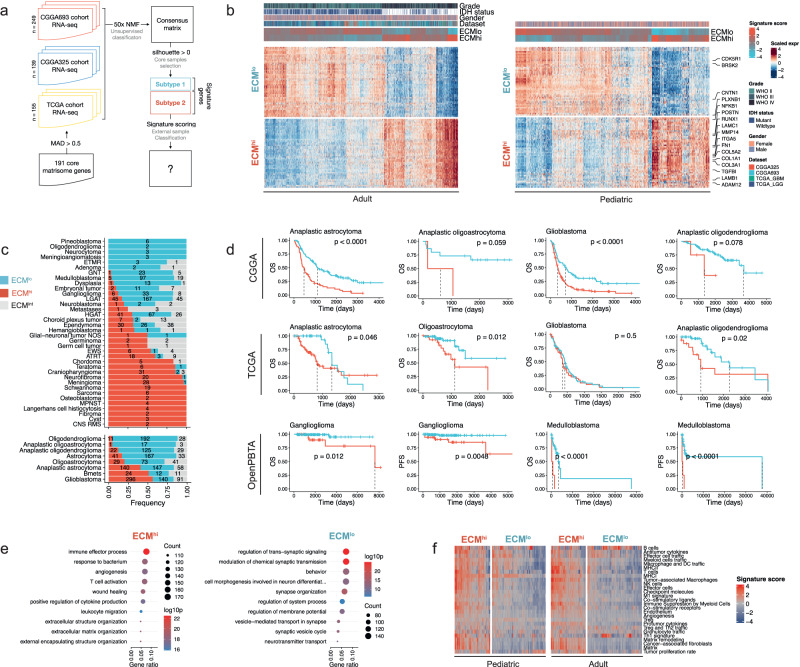
Unsupervised analysis of retrospective RNA-seq data reveals two extracellular matrix states in glioblastoma that are prognostic of clinical outcome and conserved across multiple central nervous system cancers. **a** Outline of the computational workflow. **b** ECM^hi^ and ECM^lo^ signature gene expression in adult (left) and pediatric (right) central nervous system tumors. Select signature genes are labeled. **c** Frequency of ECM states across different CNS tumors. **d** Kaplan–Meier survival curves for ECM^hi^ (red) and ECM^lo^ (blue) tumors of different histologies. Only tumors with a statistically significant effect (*p* < 0.05, log-rank test) are shown. **e** Gene ontology (GO) terms enriched in ECM^hi^ (left) and ECM^lo^ (right) states in adult gliomas. Top 10 GO terms are shown. **f** Functional gene expression signatures from Bagaev et al. (2021) in pediatric and adult brain tumors, grouped by ECM state.

Glioblastoma (GBM) tissue, unlike that of low-grade gliomas (LGGs), has been shown to display extensive and heterogeneous ECM deposition and tissue remodeling^23^. Thus, we began our analysis with an unbiased characterization of the ECM transcriptome in GBM (Fig. 1a). First, we batch corrected (Supplementary Fig. 1a) publicly available GBM RNA-sequencing (RNA-seq) profiles from three independent datasets (TCGA, CGGA-693, CGGA-325) across 558 patients and performed non-negative matrix factorization (NMF) on the core matrisome gene expression matrix, thereby limiting our analysis to genes encoding structural components of ECM^19^. Based on cophenetic correlation coefficient (Supplementary Fig. 1f) and results from hierarchical clustering (Supplementary Fig. 1d, e), we grouped the data into two classes and defined a set of 505 samples as core samples based on a positive silhouette coefficient (Supplementary Figure 1b). Bootstrap resampling of the core matrisome gene set showed that clustering results were stable to variations in the gene set when compared to a reference gene set with a similar expression distribution (Methods, Supplementary Fig. 1c).

In order to characterize the two classes/subtypes and classify external samples, we performed differential expression analysis on core samples between classes. For each class, we identified 49 signature genes as the intersection of top marker genes across the three datasets analyzed (Fig. 1b, Supplementary Data), such that differential expression of each signature gene was supported in all three datasets (Supplementary Fig. 1g). One cluster was characterized by upregulation of genes encoding fibrillar ECM proteins, including collagens (*COL5A2*, *COL1A2*, *COL3A1*, *COL1A1*, *COL6A3*, *COL5A1*, *COL6A2*, *COL8A1*) and glycoproteins (*LAMB1*, *LAMC1*, *POSTN*, *FN1*). All the signature genes encoding ECM proteins, with the exception of *COL8A1* and *LTBP2*, have been shown to be present in GBM at the protein level (Supplementary Table 1). The clusters were defined as ECM^hi^, extracellular matrix high, (48.2% of samples) and ECM^lo^, extracellular matrix low, (51.8% of samples) to reflect their distinct ECM composition.

Next, to verify the existence of ECM^hi^ and ECM^lo^ subtypes in other CNS malignancies, we scored additional RNA-seq data spanning adult low-grade (*n* = 1164) and pediatric (*n* = 977) brain tumors as well as brain metastases (Bmets, *n* = 47) for expression of ECM^hi^ and ECM^lo^ signature genes. We found 83% of adult gliomas, 80% of pediatric gliomas, and 77% of Bmets could be reliably classified into either subtype, indicating that these programs are conserved across a range of CNS malignancies beyond GBM. The remaining samples were defined by contemporaneous up- or down-regulation of ECM^hi^ and ECM^lo^ signature genes and could not be reliably classified as either ECM^hi^ or ECM^lo^. Principal component analysis (PCA) of core matrisome gene expression placed these samples between ECM^hi^ and ECM^low^ samples, suggesting that they display an intermediate ECM state (ECM^int^) (Supplementary Fig. 1h). In adult gliomas, ECM^hi^ subtype was associated with higher tumor grade, absence of IDH mutations, and mesenchymal subtype in GBM, whereas the majority of ECM^lo^ GBM tumors were of the proneural subtype (Supplementary Fig. 1k) (*P* < 2.2E-16, Chi-Square Test). Pediatric tumors exhibited a highly skewed distribution of ECM states, where a number of tumors including craniopharyngioma (86%), neurofibroma (95%), meningioma (97%), and schwannoma (100%) were classified almost exclusively as ECM^hi^, whereas glioneuronal tumors (GNT, 3%) and medulloblastomas (4%) were classified almost exclusively as ECM^lo^ (Fig. 1c), reflecting an association between the ECM subtypes and the intrinsic biology of different brain cancers. However, we did not detect an association between ECM^hi^ score and the level of malignancy of pediatric brain tumors (Supplementary Fig. 1i), suggesting that the effect of ECM stroma may be context-specific. In astrocytoma and mixed glioma tumors as well as pediatric ganglioglioma and medulloblastoma, we found an association between ECM^hi^ subtype and decreased patient survival (Fig. 1d), suggesting that ECM expression signature can be prognostic of clinical outcome in a subset of CNS tumors. In GBM, however, we detected an association in only one of the cohorts analyzed (Fig. 1d), indicating that ECM may not be prognostic of patient survival at baseline in GBM.

Next, we sought to determine if differences between ECM^hi^ and ECM^lo^ GBM are driven by changes in ECM gene expression alone, or by other gene expression programs that could induce ECM gene expression changes as a bystander effect. First, ECM subtypes were applied to primary and recurrent tumors from the longitudinal Glioma Longitudinal AnalySiS (GLASS) cohort. We did not observe any association between ECM subtype and tumor recurrence (Supplementary Fig. 1j), contrary to previous findings that ECM deposition is higher in recurrent GBM tissue^23^. Next, we performed differential expression analysis between primary ECM^hi^ and ECM^lo^ tumors. Gene ontology (GO) enrichment analysis of differentially expressed genes revealed an upregulation of immune effector process, wound healing, and angiogenesis pathways in adult glioma ECM^hi^ tumors (Fig. 1e). In contrast, ECM^lo^ samples were characterized by upregulation of neurosynaptic pathways (Fig. 1e), likely suggesting an enrichment of non-neoplastic cells. Additionally, ECM^hi^ tumors upregulated numerous immune and stromal-related signatures (Fig. 1f).

Using data from the Ivy Glioblastoma Atlas Project (Ivy-GAP), we confirmed that ECM^lo^ signature was preferentially expressed at the leading edge (LE) and infiltrative region (IT) of the tumor, which are known to consist largely of non-neoplastic cells^24^, whereas the ECM^hi^ signature was upregulated in regions corresponding to microvascular proliferation (CTmvp) and hyperplastic blood vessels (CThbv), in line with a recent study^25^ (Supplementary Fig. 1l). We also examined in situ hybridization (ISH) and adjacent hematoxylin and eosin (H&E) tissue sections annotated for the same histologic features. We found that ECM^hi^ hallmark genes *COL1A1*, *COL4A1* were expressed in CTmvp regions (Supplementary Fig. 1m), suggesting that ECM^hi^ signature is spatially associated with GBM vasculature.

### ECM^hi^ tumors are characterized by the presence of perivascular fibroblasts whose enrichment predicts poor response to immunotherapy

**Fig. 2:**
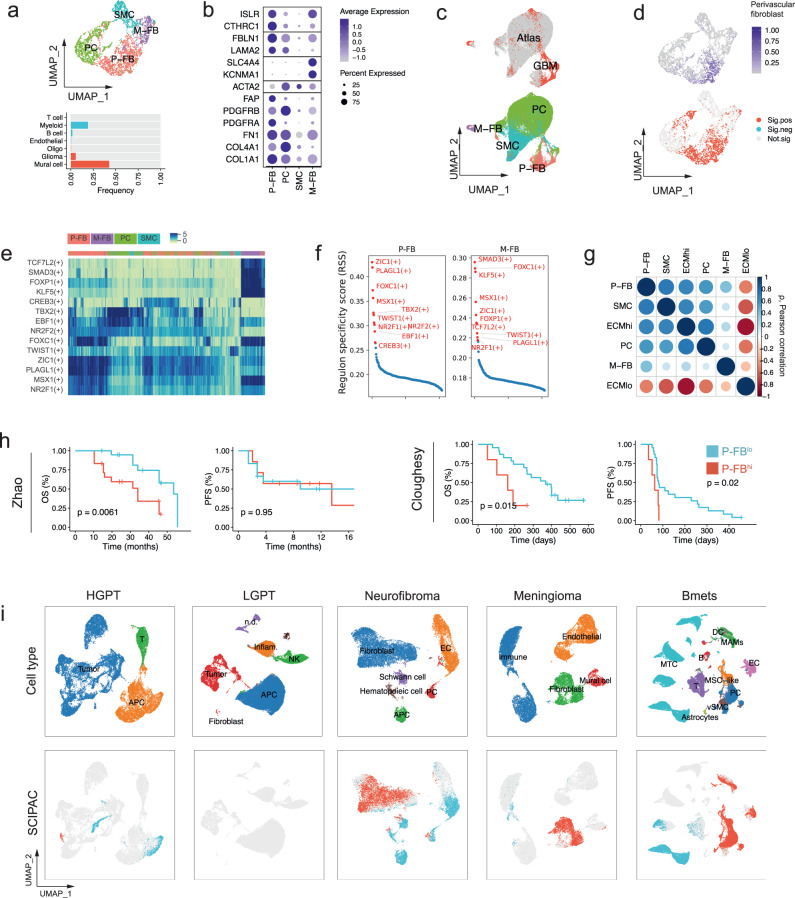
Cancer associated fibroblast-like cells of perivascular origin are present in ECM^hi^ GBM and predict poor response to immunotherapy. **a** Top: a UMAP plot showing subpopulations of mural cells. Bottom: barplot showing the frequency of ECM^hi^-associated cells within each cell type. **b** Marker genes identifying subpopulations of mural cells in GBM. P-FB – perivascular fibroblast; PC – pericyte; SMC – smooth muscle cell; M-FB – meningeal fibroblast. **c** Joint UMAP embedding of mural cells from a human brain vascular atlas (grey) and GBM mural cells (red). **d** UMAP embedding of GBM mural cells, colored by the enrichment of the perivascular fibroblast metagene (top) and SCIPAC predictions (bottom). **e** SCENIC-inferred regulon activity in subpopulations of mural cells. Shown in red are top 10 regulons for each mural cell subpopulation based on regulon specificity score (RSS). **f** RSS of transcription factors in perivascular and meningeal fibroblasts; 10 transcription factors with the highest RSS are labeled in red. **g** Pearson correlation between ECM^hi^/ECM^lo^ metagenes and mural cell subpopulations. **h** Kaplan–Meier plot of overall and progression free survival for patients with high (red) or low (blue) presence of perivascular fibroblasts in Cloughesy (*n* = 28) and Zhao (*n* = 38) cohorts treated with anti-PD1 therapy (P values were calculated using log-rank test). **i** UMAP embeddings of scRNA-seq data from different tumors, colored by cell type (top) and positive (red) or negative (blue) association with ECM^hi^ state, as predicted by SCIPAC. HGPT – high-grade pediatric tumors; LGPT – low-grade pediatric tumors.

The vascular microenvironment is an important brain tumor niche with a heterogeneous and not fully revealed cellular makeup^26^. In order to identify vascular/perivascular cellular components contributing to the ECM stroma in CNS tumors, we applied SCIPAC – a tool designed to identify phenotype-associated cells in single-cell RNA-sequencing (scRNA-seq) data – to a scRNA–seq dataset from 16 GBM patients (Supplementary Fig. 2a–e)^27^. SCIPAC predicted 43% of *PDGFRβ* + *ACTA2*+ mural cells to be associated with the ECM^hi^ signature (Fig. 2a). We sub-clustered and annotated mural cells based on gene expression signatures characterizing previously identified perivascular cell types in the human brain^9^. We identified two clusters as perivascular fibroblasts (P-FB; *FBLN1*, *LAMA2*) and meningeal fibroblasts (M-FB; *SLC4A4*, *KCNMA1*), as well as separate clusters of pericytes (PC; *PDGFRB*, *COL4A1*), and smooth muscle cells (SMC; *ACTA2*) (Fig. 2b). Integration of GBM mural cells with those from a human brain vascular atlas^9^ resulted in alignment of respective subpopulations in UMAP space (Fig. 2c). We found that 86% of perivascular fibroblasts were associated with the ECM^hi^ phenotype, suggesting their pro-fibrotic role in the GBM TME (Fig. 2c). Additionally, we found that perivascular fibroblasts resembled previously identified murine brain fibroblast-like cells^28^ (Supplementary Fig. 2i). To determine the contribution of these perivascular cells to ECM^hi^ and ECM^low^ states, we deconvoluted bulk gene expression profiles using a set of signature genes identifying each perivascular cell type. We found that ECM^hi^ metagene was highly correlated to perivascular fibroblast, pericyte, and SMC signatures but not to meningeal fibroblast signature (Fig. 2g), likely reflecting their low frequency in GBM, which is consistent with rare cases of primary extracerebral meningeal GBM^29^. Notably, the P-FB cluster expressed several classical CAF markers such as *PDGFRA*, *PDGFRB*, *COL1A1*, and *FAP*^17,30,31^. Strikingly, we found that enrichment of perivascular fibroblasts was prognostic of poor clinical outcome in GBM (Supplementary Fig. 2g) and correlated with mesenchymal subtype (Supplementary Fig. 2f), suggesting their possible pro-tumorigenic role. Therefore, we refer to this population of cells as “CAF-like” whenever appropriate to reflect their CAF-like tumor-promoting phenotype.

Next, we applied SCENIC to infer active regulators of the fibroblast populations and confirm their identity in GBM. For perivascular and meningeal fibroblast populations, SCENIC predicted a high activity of several common brain fibroblast transcription factors ZIC1, FOXC1, NR2F2^32^, TWIST1^33^, and a CAF-specific transcription factor NR2F1 (Fig. 2f)^34^. The activity of these regulons was restricted to cells with perivascular and meningeal fibroblast signatures, indicating that their phenotype is distinct from that of pericytes or smooth muscle cells (Fig. 2e). These results suggest that fibrotic scarring in GBM may share some of its mechanisms with other CNS pathologies in which fibrogenic cells are derived from perivascular fibroblasts in response to inflammatory stimuli. Interestingly, however, our analysis of genomic copy number using the CopyKat algorithm identified a fraction of perivascular stromal cells as aneuploid (Supplementary Fig. 2d). Since mesenchymal differentiation of glioma stem cells (GSCs) into pericytes has been previously shown (deCarvalho et al. 2010; Ricci-Vitiani et al. 2010), these findings raise an intriguing possibility that malignant cells can undergo a mesenchymal differentiation to assume a CAF-like phenotype in GBM.

Next, to identify the presence of closely related stromal cell types in other ECM^hi^-enriched CNS tumors, we analyzed scRNA-seq data from neurofibroma (*n* = 3), meningioma (*n* = 7), low-grade pediatric tumors (LGPT; *n* = 26), high-grade pediatric tumors (HGPT; *n* = 23) and Bmets (*n* = 15) (Fig. 2i). Stromal/mesenchymal cells were detected at the highest frequency in BMets (21%), meningioma (26%), and neurofibroma (56%). We did not detect any stromal cells in HGPTs which our previous analysis identified to be ECM^hi^-enriched which could be ascribed to variability in cell isolation protocols, since detachment of cells embedded in the basement membrane requires stronger tissue dissociation methods^28,35^.

In order to verify their fibrogenic phenotype, we applied SCIPAC on scRNA-seq and tumor-matched bulk RNA-seq data. SCIPAC predicted fibroblasts as the cellular source of ECM in neurofibroma and meningioma (Fig. 2i). In Bmets, in addition to perivascular stromal cells, myeloid cells and endothelial cells were significantly associated with ECM^hi^ phenotype, suggesting their possible role in ECM remodeling. No ECM^hi^-associated cells were found in HGPT and LGPT tumors, consistent with the absence of stromal cell types in these datasets. Next, in order to identify conserved stromal cell states across different cancers, we performed the mutual nearest neighbors (MNN) batch correction on combined data and sub-clustered stromal cells based on MNN-corrected gene expression values (Supplementary Fig. 2j, k). The majority of GBM perivascular fibroblasts were clustered together with mesenchymal stem-like cells (MSCs) from Bmets. Indeed, perivascular CAF-like cells also upregulated mesenchymal progenitor markers *CTHRC1* and *ISLR* (Fig. 2d)^36^, indicating that MSCs may be a source of CAF-like cells in GBM, as previously suggested^14^. Neurofibroma fibroblasts, which have been characterized as distinct from classical fibroblasts^37^, formed a separate cluster. Clusters 3, 4, and 5 predominantly contained meningioma mesenchymal subtypes together with meningeal GBM fibroblasts; cluster 0 consisted almost entirely of pericytes and smooth muscle cells. Overall, the results of this integrative analysis suggest that despite their common role in ECM remodeling of the tumor stroma, stromal cells exhibit distinct and largely non-overlapping phenotypes across different CNS malignancies.

Finally, to determine whether perivascular fibroblasts play a role in anti-tumor immunity and response to immunotherapy in GBM, we interrogated retrospective data from two recent anti-PD1 immunotherapy trials^21,22^. In both cohorts, we found a significant reduction in overall survival for patients whose tumors were enriched in perivascular fibroblasts (Fig. 2h), but not other mural cell subpopulations (Supplementary Fig. 2h), suggesting that the presence of perivascular fibroblasts is prognostic of a poor immunotherapeutic response.

### Glioblastoma perivascular fibroblasts express chemotactic factors that may recruit tumor-associated macrophages to the TME

**Fig. 3:**
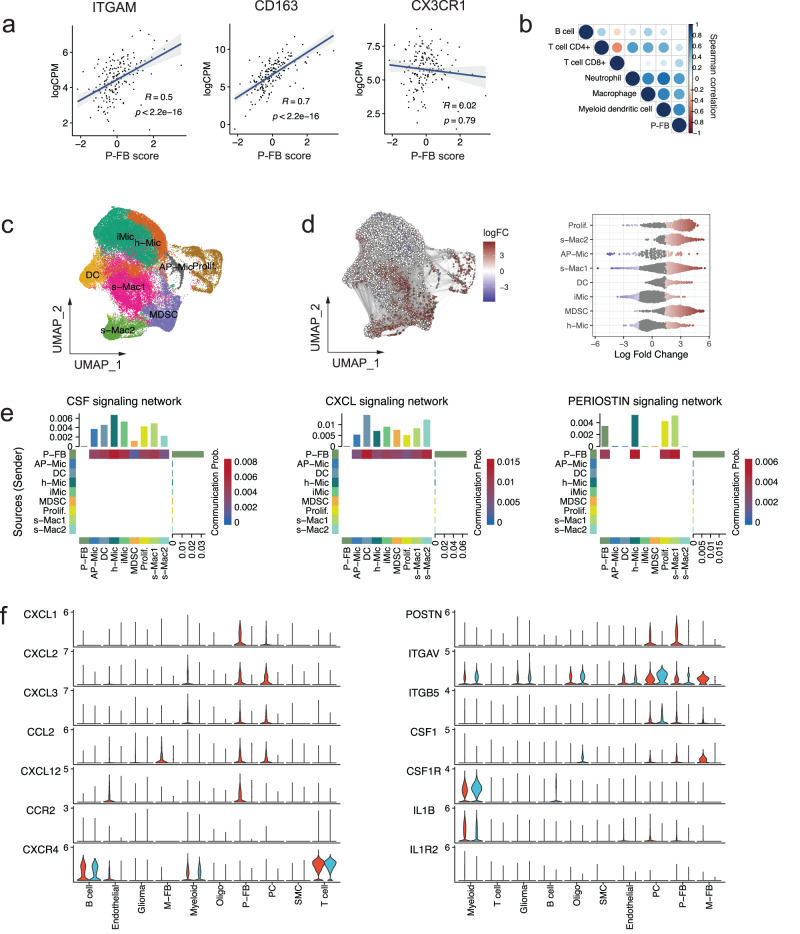
Perivascular fibroblasts express chemotactic factors that may recruit tumor-associated macrophages to the GBM TME. **a** Correlation between perivascular fibroblast (P-FB) signature score and monocyte-derived macrophage (*ITGAM*, *CD163*) and microglial (*CX3CR1*) marker genes. Value of the spearman correlation coefficient is shown together with the *p* value. **b** Spearman correlation between perivascular fibroblast signature score and immune cells fractions in bulk RNA-seq data, estimated using the TIMER algorithm. **c** UMAP embedding of myeloid cells colored by cell subpopulation. **d** Differential abundance analysis of ECM^hi^ and ECM^lo^ tumors. The left panel shows a graph of cellular neighborhoods superimposed on the UMAP embedding of the data. Color represents log fold change (logFC) relative to ECM^lo^, node size represents the size of the neighborhood, and edge width represents the amount of overlap between neighborhoods. Relative changes which were not statistically significant (*p* > 0.05) are not shown. The right panel shows the distribution of differential abundance of cellular neighborhoods across myeloid subpopulations. **e** Heatmaps showing interaction strength between P-FB cells and myeloid cells for select pathways. Mac – macrophage; Mic – microglia; Prolif. – proliferating macrophage; MDSC – myeloid-derived suppressor cell; DC – dendritic cell. **f** Expression of selected ligands and their receptors for pathways from (**e**).

Next, we hypothesized that GBM perivascular fibroblasts may play a role in modulating neuroinflammation and contribute to the establishment of an immunosuppressive TME that is resistant to immune checkpoint blockade such as anti-PD1 antibodies. To verify this, we first performed cell type deconvolution of GBM bulk gene expression profiles. We found a strong positive correlation between the presence of perivascular fibroblasts and myeloid cells, including macrophages, dendritic cells, and monocytes (Fig. 3a). Perivascular fibroblast signature was positively correlated to expression of CD11B (*ITGAM*) and *CD163*, but not *CX3CR1* (Fig. 3b), indicative of increased numbers of monocyte-derived macrophages^38^, as well as T cell exhaustion markers (Supplementary Fig. 3a). This was supported by differential abundance analysis of GBM scRNA-seq data, in which we found an enrichment of two populations of tumor-associated macrophages (TAMs) s-Mac1 and s-Mac2 (Supplementary Figure 2b), myeloid-derived suppressor cells (MDSCs) and proliferating (Prolif.) macrophages in samples that were classified as ECM^hi^ based on pseudobulk expression profiles (Fig. 3c, Supplementary Fig. 2b, c). Additionally, macrophages, dendritic cells (DCs) and MDSCs displayed a lower expression of genes encoding MHC class II (MHC-II) molecules (Supplementary Fig. 3b), indicating their poor antigen-presenting capacity, characteristic of the M2-like macrophage state.

To investigate possible mechanisms of macrophage recruitment by perivascular fibroblasts, we applied the CellChat algorithm that can infer cell-state specific signaling communications from scRNA-seq data^39^. CellChat predicted active chemoattractant signaling from perivascular fibroblasts to macrophages via chemokines CCL2, CXCL1, CXCL2, CXCL12, CSF1, and matricellular protein periostin (POSTN), which are known to induce chemotaxis and alternative polarization of tumor-supporting M2-like myeloid cells (Fig. 3d–f)^3,40–44^. These results suggest that perivascular fibroblasts may contribute to the establishment of an immunosuppressive microenvironment by driving M2-like TAM recruitment and polarization via chemotaxis and periostin signaling, respectively.

### Perivascular fibroblasts promote immune-evasive stem-like cancer cell phenotype in GBM

**Fig. 4:**
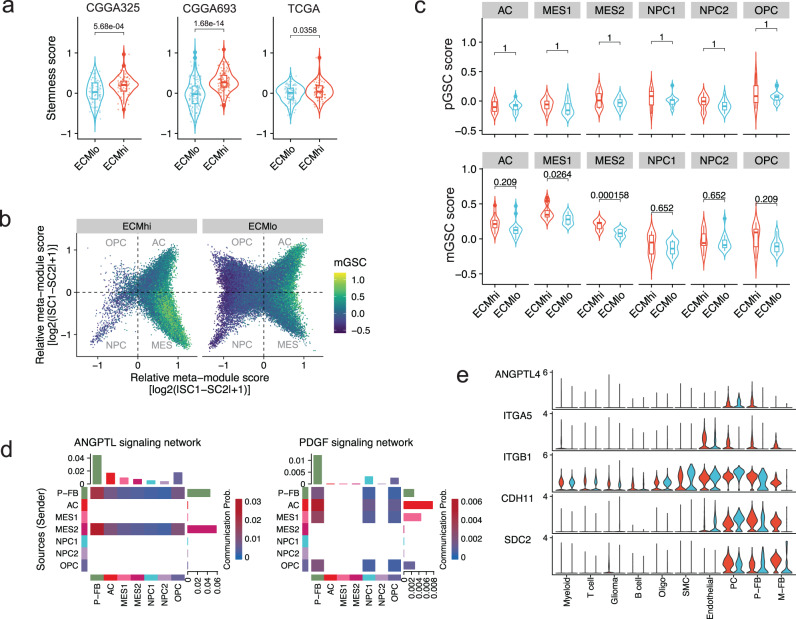
Perivascular fibroblasts secrete factors that upregulate stem-like programs in ECM^hi^ glioblastoma. **a** Stemness signature scores in ECM^hi^ and ECM^lo^ tumors in TCGA (*n* = 175), CGGA325 (*n* = 325) and CGGA693 (*n* = 693) GBM cohorts. **b** Two-dimensional butterfly plot of glioma cell states in ECM^hi^ and ECM^lo^ tumors, colored by enrichment of the mesenchymal glioma stem cell (mGSC) signature. OPC – oligodendrocyte progenitor cell-like; AC astrocyte-like; NPC – neural progenitor cell-like; MES – mesenchymal-like. **c** Proneural (top) and mesenchymal (bottom) glioma stem cell scores in ECM^hi^ and ECM^lo^ tumors. Two-sided t-test. Benjamini-Hochberg adjusted *P* values are shown. **d** Strength of outgoing and incoming signaling in perivascular fibroblasts (P-FB) and MES-like glioma cells for selected pathways with known role in cancer stem cell maintenance. **e** Expression of ANGPTL4 and its receptors in GBM scRNA-seq data. In (**a**) and (**c**), *p* values were obtained using two-tailed t-test. P values corrected for multiple comparisons using the Holm–Bonferroni method are shown. For violin plots in (**a**) and (**c**), the center lines represent the median. The lower and upper hinges correspond to the first and third quartiles (the 25th and 75th percentiles, respectively). The upper whisker extends from the hinge to the largest value no further than 1.5 times of inter-quartile range (IQR) from the hinge. The lower whisker extends from the hinge to the smallest value at most 1.5 times of IQR from the hinge.

GSCs are maintained within perivascular collagen-rich niches^45,46^, and are known to evade antitumor immune responses through various mechanisms, including downregulation of MHC class I, induction of quiescence, and other mechanisms that promote immune tolerance^47^. In GBM, CAFs are known to enrich GSCs^14^. Therefore, we hypothesized that GBM perivascular fibroblasts may also modulate anti-tumor immune responses through maintenance of GSCs in the perivascular niche. In order to verify this, we first quantified the stem cell-like tumor phenotype by computing “stemness score”^48^. We found that ECM^hi^ tumors displayed a higher stemness score across all three datasets analyzed (Fig. 4a), suggesting that a fibrotic microenvironment may favor the emergence and/or maintenance of glioma stem-like cell phenotype. We then classified glioma cells into oligodendrocyte-progenitor-like (OPC-like), neural-progenitor-like (NPC-like), astrocyte-like (AC-like), and mesenchymal-like (MES-like) cell states^49^, and found that ECM^hi^ tumors were characterized by an enrichment of MES-like cells and reduced frequency of NPC-like and OPC-like states (Fig. 4b, Supplementary Fig. 3d), which is in line with the preponderance of myeloid cells in ECM^hi^ tumors and their proposed role in maintaining the MES-like cell state^49^.

GBM contains hierarchies of mesenchymal and proneural GSCs (mGSCs and pGSCs, respectively) that are considered largely responsible for cancer cell heterogeneity observed within GBM tumors^50^. To understand the contribution of these progenitor states to glioma cell heterogeneity observed in ECM^hi^ and ECM^low^ subtypes, we scored single cells using previously identified mGSC and pGSC signature genes. We found that MES-like glioma cells in ECM^hi^ tumors were enriched in mGSCs (Fig. 4b, S4e), which is in line with their increased frequency.

Next, to elucidate possible mechanisms of mGSC enrichment, we compared CellChat-inferred signaling networks between ECM^hi^ and ECM^lo^ tumors. We found an upregulation of ANGPTL and PDGF signaling by perivascular fibroblasts to glioma cells, which have been implicated in maintenance of stem-like cell phenotype in GBM (Fig. 4d)^51–53^. MES-like glioma cells expressed multiple receptors for ANGPTL4, including SDC2 SDC3, SDC4, and integrins ITGA5 and ITGB1 (Fig. 4e), suggesting that the ANGPTL pathway may be responsible for the enrichment of GSCs in ECM^hi^ tumors.

## Discussion

Previous studies have shown that therapeutically reducing ECM deposition can alleviate immunosuppression across various cancer models, including GBM^54–56^. However, to date, comprehensive analyses of the ECM across different brain tumors, which could provide important insights into tumor progression and treatment resistance, have been limited. In this study, we undertake a pan-CNS analysis of the ECM transcriptome to identify which cellular and molecular components contribute to the formation of ECM stroma in CNS tumors. Using unsupervised analysis of core matrisome gene expression in 558 GBM tumors, we define two ECM subtypes that capture up- or down-regulation of ECM gene expression and are conserved across at least 20 additional adult and pediatric CNS cancers in over 2000 tumor samples. In this study, we find an association between the ECM subtypes and the intrinsic biology of different brain cancers as well as their clinical course and therapy response.

While our study presents new findings through its purely computational approach, it is crucial to acknowledge its inherent limitations. First, despite our efforts to computationally validate our findings, ECM signatures discovered here may represent technical or regional artifacts rather than true inter-tumoral heterogeneity. Therefore, future studies should experimentally investigate whether ECM proteins that define ECM^hi^ subtype co-exist within the same tumor, and whether variable abundance of these proteins also gives rise to the ECM^hi/lo^ dichotomy on the proteomic level.

Deposition of ECM components in brain tumors has been proposed to have three different origins: adjacent stromal cells, normal brain cells that are activated in response to tumor-derived factors^57^, or by malignant cells as a part of their mesenchymal phenotype^58^. In human GBM specimens, ECM-rich zones bordering cellular tumor regions and around blood vessels are characterized by high expression of smooth muscle actin^23^ (encoded by *ACTA2*) – a marker of the pericyte lineage in the normal brain, suggesting the contribution of perivascular stromal cells. A recent study of human GBM identified pericytes as the cellular source of an ECM gene signature with a negative prognostic value at recurrence^25^. However, the reliance of this study on cell surface markers without comprehensive gene expression profiling raises the possibility that the identified PDGFRβ+ cells could be other cells in the microenvironment such as fibroblasts or smooth muscle cells, which share overlapping cell surface markers with pericytes^59^. Indeed, Dias et al. identified a subpopulation of *Slc1a3*+ pericytes as the main source of scar-forming αSMA+ cells in mouse models of traumatic brain injury, ischemic stroke, and multiple sclerosis, but not glioma^11^.

Our findings similarly implicate GBM vasculature in ECM deposition. ECM^hi^ subtype identified in this study is characterized by overexpression of genes encoding cerebrovascular ECM proteins such as collagens, laminins, and fibronectin (Lau et al. 2013) and is enriched in the perivascular tumor region. Our results suggest that perivascular fibroblasts may be primarily responsible for ECM deposition in GBM and may therefore act as analogs of CAFs in peripheral tumors. Recent efforts to comprehensively characterize human cerebrovasculature identified and characterized this cell population in human and murine brains^9,28,35,60^. The presence of perivascular fibroblasts in GBM vasculature has been reported previously^61^, and a recent study by Jain et al. reported isolation of cells with phenotypic and morphological similarities to CAFs. Although the cellular origin of these diploid cells is unknown, it is plausible that these cells originate from perivascular CNS fibroblasts that become recruited and activated in response to tumor-derived factors. While we cannot conclude that the cells identified in our analysis represent bona fide CAFs based solely on in silico results, perivascular fibroblast population expresses several CAF-specific gene markers (*PDGFRA*, *FAP*, *FN1*, *COL1A1*) and transcription factors (*NR2F1*) (Wu et al. 2022), which warrants further investigation of their identity and pro-tumorigenic roles in GBM.

Interestingly, we find that about 25% of cells in the P-FB population carry the same genomic abnormalities as malignant glioma cells, indicating that at least some fraction of perivascular fibroblasts may have a neoplastic origin. GSCs are known to localize to perivascular niches and have the ability to undergo mesenchymal differentiation^62,63^. Indeed, an analysis of human GBM specimens showed that GSCs can generate vascular pericytes upon stimulation with TGF-β, and that the majority of GBM pericytes are derived from malignant cells^64^. Whether GSCs can directly assume a fibroblast identity is still unknown; however, pericytes have been shown to undergo differentiation into fibroblasts upon detachment of tumor microvasculature^65^, as well as in kidney fibrosis^66^. Therefore, our findings raise an intriguing possibility that a fraction of GBM perivascular fibroblasts may originate from GSCs.

In epithelial tumors such as breast, colon, and pancreatic carcinomas, CAFs are known to suppress effector immune cell activation and tumor infiltration, leading to resistance to immunotherapies such as ICB^3^. Our findings suggest that perivascular fibroblasts, similar to CAFs in peripheral tumors, may play a role in immunosuppression and immunotherapy resistance. Our results show that perivascular fibroblasts may simultaneously induce GSCs and reprogram the immune response to facilitate tumor immune evasion and immunotherapy resistance. GSCs are known to be less immunogenic, evade immune responses through the downregulation of MHC molecules and promote immune tolerance^47^. We also show that perivascular CAF-like cells can activate the production of chemoattractant molecules to recruit M2-like macrophages into the GBM TME, which contributes to immunosuppression. In addition to attraction and retention of tumor-promoting myeloid cells, CAFs are known to affect antitumor immune responses indirectly by production and remodeling of ECM components, which serves as a physical barrier restricting access of immune cells to cancer cells. Indeed, a recent immunohistochemical analysis of GBM tissue showed an enrichment of T cells in ECM-rich zones^23^, suggesting that T cell trafficking to the tumor is impeded due to pathologically high deposition of ECM components by stromal cells in the perivascular niche. This has important implications for adoptive cell-based therapies, such as CAR-T and CAR-NK cell therapies. CAR-T cell therapy in GBM has shown limited success, partly due to limited CAR-T cell infiltration into the tumor when CAR-T cells are administered systemically^67,68^. This suggests that overcoming these biophysical and biochemical barriers by targeting perivascular fibroblasts in GBM may help overcome immunotherapy resistance and increase patient survival.

## Methods

### scRNA-seq data processing and analysis

The scRNA-seq data (GSE182109) was obtained from Gene Expression Omnibus^27^. Individual samples were log-normalized and integrated using Seurat’s (v4.3.0.1) reciprocal PCA^69^. Doublets were identified and removed using Scrublet (v0.2.3) algorithm^70^. Next, cells were clustered and annotated based on previously reported marker genes^27^ as well as copy number alterations (CNAs) inferred using CopyKat (v1.1.0) algorithm^71^. To exclude poor quality cells within each cell type, we applied median absolute deviation (MAD)-based outlier detection approach, as described previously^72^. Clusters characterized by low UMI counts, high fraction of mitochondrial reads, and uninformative marker genes were removed.

Malignant cells were assigned to meta-modules defined by Neftel et al. Briefly, single-cell scores were computed for each of the signatures (MES1-like, MES2-like, NPC1-like, NPC2-like, AC-like, OPC-like), and cells were assigned to each cell state, as previously described^49^.

### RNA-seq data processing and analysis

Publicly available RNA-seq data were downloaded from TCGA and CGGA databases. In total, three cohorts were used in this study: TCGA GBM, CGGA-693, and CGGA-325 (Supplementary Table 2). The datasets were TMM-normalized (edgeR v3.40.2), and logCPM values were used for all downstream analyses. The datasets were combined, and batch-corrected using limma’s (v3.54.2) *removeBatchEffect* function.

Spatial distributions of ECM signatures were examined using the Ivy GAP dataset^24^. Gene expression FPKM values were log transformed prior to analysis.

### Identification and validation of extracellular matrix subtypes

Batch-corrected logCPM values were mean-centered prior to clustering. Using the core matrisome geneset NABA_CORE_MATRISOME available in the Molecular Signature Database (MSigDB), we performed consensus non-negative matrix factorization (NMF) with rank 2 using NMF R package (v0.26), as we observed the highest average cophenetic correlation coefficient for *k* = 2. We also performed consensus hierarchical clustering using the ConsensusClusterPlus package (v1.62.0) with average linkage which produced similar clustering results^73^.

To determine how stable our clustering results are to variations in the gene set, and if ECM^hi^ or ECM^lo^ states can be recapitulated by other gene sets, we performed bootstrap resampling on the core matrisome geneset and compared our clustering outcomes to each resampled dataset using the Adjusted Rand Index (ARI). To generate a null ARI distribution, we performed NMF using a control gene set by binning all analyzed genes into 30 bins based on average expression in all samples, and for each gene in the core matrisome gene set, randomly selecting a gene from the same expression bin.

In order to characterize ECM signatures and classify external samples, we generated subtype-specific gene signatures by searching for genes that were upregulated in all three datasets, according to the Wilcoxon test. As a result, a set of 49 signature genes for each subtype was obtained.

### Classification of external samples

External samples were classified into subtypes/signatures based on their signature scores, which were computed as previously described^49^. Since signature scores reflect up- or down-regulation of a gene signature compared to a control geneset, we classified samples into subtypes based on the sign of the signature score. For example, Samples scoring greater than 0 for gene signature of subtype A and lower than 0 for the signature of subtype B were classified as subtype A, samples scoring greater than 0 for gene signature of subtype B and lower than 0 for the signature of subtype A were classified as subtype B, and other samples are labeled as unidentified. For scRNA-seq data, pseudo bulk samples were generated, TMM-normalized, and classified as outlined above.

### Wang subtype classification

Uncorrected bulk RNA-seq data were classified into three molecular subtypes using the SubtypeME tool in the GlioVis portal^74,75^. Each sample was assigned to a molecular subtype with the lowest *p*-value.

### Computation of cell-type-specific gene signatures

To estimate the enrichment of cell type-specific signature genes in bulk RNA-seq data, top marker genes were first identified for a cell type/state of interest using the FindMarkers function in the Seurat package^69^. Genes expressed in fewer than 50% of cells and with fold change (FC) less than 2 were removed. Top 50 genes with the highest log2FC were considered as top marker genes. Bulk tumors were then scored for the resulting top marker genes as previously described^49^. Samples were classified as high or low for a cell type/state, if their signature score fell into the upper or lower quartiles, respectively. For immunotherapy cohorts, signature score of 1 was used as a cutoff.

### Reporting summary

Further information on research design is available in the Nature Research Reporting Summary linked to this article.

### Supplementary information

Supplementary Information

REPORTING SUMMARY

## Data Availability

No new datasets were generated as a part of this study. Accession codes, unique identifiers, or web links for publicly available datasets are provided in Supplementary Table 2.

## Code availability

Code supporting the current study has been deposited on GitHub: https://github.com/plezar/NPJ-GBM-PFB_code.
