# Supplementary material for: CNS tumor stroma transcriptomics identify perivascular fibroblasts as predictors of immunotherapy resistance in glioblastoma patients
Source: NPJ Genom Med. 2023 Oct 26;8:35. doi: 10.1038/s41525-023-00381-w (PMC10603041; doi:10.1038/s41525-023-00381-w)
Supplement: Supplementary file 1 — Supplementary Information [file 41525_2023_381_MOESM1_ESM.pdf]

Supplementary Table 1. Studies reporting protein expression data for $ECM^{hi/lo}$ signature genes that encode extracellular matrix (ECM) components.

Gene	Signature	Classification	Category	Protein	Reference	Detected
LAMB1	ECM^{hi}	Core matrisome	ECM Glycoproteins	laminin, beta 1	[1]	+
COL5A2	ECM^{hi}	Core matrisome	Collagens	collagen, type V, alpha 2	[2]	+
COL1A2	ECM^{hi}	Core matrisome	Collagens	collagen, type I, alpha 2		
COL3A1	ECM^{hi}	Core matrisome	Collagens	collagen, type III, alpha 1	[3]	+
LAMC1	ECM^{hi}	Core matrisome	ECM Glycoproteins	laminin, gamma 1 (formerly LAMB2)	[1]	+
SERPINH1	ECM^{hi}	Matrisome-associated	ECM Regulators	serpin peptidase inhibitor, clade H (heat shock protein 47), member 1, (collagen binding protein 1)	[2]	+
PLOD1	ECM^{hi}	Matrisome-associated	ECM Regulators	procollagen-lysine 1, 2-oxoglutarate 5-dioxygenase 1	[4]	+
MMP14	ECM^{hi}	Matrisome-associated	ECM Regulators	matrix metallopeptidase 14 (membrane-inserted)	[5]	+
COL1A1	ECM^{hi}	Core matrisome	Collagens	collagen, type I, alpha 1	[6]	+
COL6A3	ECM^{hi}	Core matrisome	Collagens	collagen, type VI, alpha 3	[7]	+
COL5A1	ECM^{hi}	Core matrisome	Collagens	collagen, type V, alpha 1	[8]	+
SRPX2	ECM^{hi}	Core matrisome	ECM Glycoproteins	sushi-repeat-containing protein, X-linked 2	[9]	+
COL6A2	ECM^{hi}	Core matrisome	Collagens	collagen, type VI, alpha 2	[10]	+
ADAM12	ECM^{hi}	Matrisome-associated	ECM Regulators	ADAM metallopeptidase domain 12	[11]	+
POSTN	ECM^{hi}	Core matrisome	ECM Glycoproteins	periostin, osteoblast specific factor	[1]	+
LTBP2	ECM^{hi}	Core matrisome	ECM Glycoproteins	latent transforming growth factor beta binding protein 2	[2]	-

SERPINE1	ECM^{hi}	Matrisome-associated	ECM Regulators	serpin peptidase inhibitor, clade E (nexin, plasminogen activator inhibitor type 1), member 1	[12]	+
LOXL2	ECM^{hi}	Matrisome-associated	ECM Regulators	lysyl oxidase-like 2	[13]	+
TGFBI	ECM^{hi}	Core matrisome	ECM Glycoproteins	transforming growth factor, beta-induced, 68kDa	[14]	+
FN1	ECM^{hi}	Core matrisome	ECM Glycoproteins	fibronectin 1	[15,16]	+
THBS1	ECM^{hi}	Core matrisome	ECM Glycoproteins	thrombospondin 1	[17,18]	+
ANXA2	ECM^{hi}	Matrisome-associated	ECM-affiliated Proteins	annexin A2	[1]	+
COL8A1	ECM^{hi}	Core matrisome	Collagens	collagen, type VIII, alpha 1	[2]	-
PLXNB1	ECM^{lo}	Matrisome-associated	ECM-affiliated Proteins	plexin B1	[2]	+

Supplementary Table 2. Datasets used in the study with links to data sources.

Dataset	Link	Reference
scRNA-seq of pediatric high-grade gliomas	https://scpca.alexslemonade.org/projects/SCPCP000001	[19]
scRNA-seq of pediatric low-grade gliomas	https://scpca.alexslemonade.org/projects/SCPCP000002	[20]
scRNA-seq of meningioma	https://zenodo.org/record/6473604	[21]
scRNA-seq of neurofibroma	https://www.ncbi.nlm.nih.gov/geo/query/acc.cgi?acc=GSE163028	[22]
scRNA-seq of brain metastases	https://www.ncbi.nlm.nih.gov/geo/query/acc.cgi?acc=GSE186344	[23]
scRNA-seq of normal human cerebrovasculature	https://www.ncbi.nlm.nih.gov/geo/query/acc.cgi?acc=GSE163577	[24]
scRNA-seq of glioblastoma and low-grade glioma	https://www.ncbi.nlm.nih.gov/geo/query/acc.cgi?acc=GSE182109	[25]
CGGA	http://www.cgga.org.cn/	[26]
TCGA	https://www.cancer.gov/ccg/research/genome-sequencing/tcga	[26]
The Glioma Longitudinal Analysis Consortium (GLASS)	https://www.synapse.org/#!Synapse:syn17038081/wiki/585622	[27]

References

1. Sethi, M. K. *et al.* In-Depth Matrisome and Glycoproteomic Analysis of Human Brain Glioblastoma Versus Control Tissue. *Mol. Cell. Proteomics* **21**, 100216 (2022).
2. Uhlén, M. *et al.* Proteomics. Tissue-based map of the human proteome. *Science* **347**, 1260419 (2015).
3. Gao, Y., Zhu, T., Chen, J., Liu, L. & Ouyang, R. Knockdown of collagen α-1(III) inhibits glioma cell proliferation and migration and is regulated by miR128-3p. *Oncol. Lett.* **16**, 1917–1923 (2018).
4. Wang, Z., Shi, Y., Ying, C., Jiang, Y. & Hu, J. Hypoxia-induced PLOD1 overexpression contributes to the malignant phenotype of glioblastoma via NF-κB signaling. *Oncogene* **40**, 1458–1475 (2021).
5. Kasten, B. B. *et al.* Targeting MMP-14 for dual PET and fluorescence imaging of glioma in preclinical models. *Eur. J. Nucl. Med. Mol. Imaging* **47**, 1412–1426 (2020).
6. Comba, A. *et al.* Spatiotemporal analysis of glioma heterogeneity reveals COL1A1 as an actionable target to disrupt tumor progression. *Nat. Commun.* **13**, 3606 (2022).
7. Cescon, M. *et al.* Collagen VI sustains cell stemness and chemotherapy resistance in glioblastoma. *Cell. Mol. Life Sci.* **80**, 233 (2023).
8. Tsai, H.-F. *et al.* Type V collagen alpha 1 chain promotes the malignancy of glioblastoma through PPRC1-ESM1 axis activation and extracellular matrix remodeling. *Cell Death Discov* **7**, 313 (2021).
9. Tang, H. *et al.* SRPX2 Enhances the Epithelial–Mesenchymal Transition and Temozolomide Resistance in Glioblastoma Cells. *Cell. Mol. Neurobiol.* **36**, 1067–1076 (2016).

10. Zhu, J., Lin, Q., Zheng, H., Rao, Y. & Ji, T. The pro-invasive factor COL6A2 serves as a novel prognostic marker of glioma. *Front. Oncol.* **12**, 897042 (2022).

11. Kodama, T. *et al.* ADAM12 is selectively overexpressed in human glioblastomas and is associated with glioblastoma cell proliferation and shedding of heparin-binding epidermal growth factor. *Am. J. Pathol.* **165**, 1743–1753 (2004).

12. Vachher, M., Arora, K., Burman, A. & Kumar, B. NAMPT, GRN, and SERPINE1 signature as predictor of disease progression and survival in gliomas. *J. Cell. Biochem.* **121**, 3010–3023 (2020).

13. Du, X.-G. & Zhu, M.-J. Clinical relevance of lysyl oxidase-like 2 and functional mechanisms in glioma. *Onco. Targets. Ther.* **11**, 2699–2708 (2018).

14. Peng, P. *et al.* TGFBI secreted by tumor-associated macrophages promotes glioblastoma stem cell-driven tumor growth via integrin αvβ5-Src-Stat3 signaling. *Theranostics* **12**, 4221–4236 (2022).

15. Serres, E. *et al.* Fibronectin expression in glioblastomas promotes cell cohesion, collective invasion of basement membrane in vitro and orthotopic tumor growth in mice. *Oncogene* **33**, 3451–3462 (2014).

16. Gong, J., Wang, Z.-X. & Liu, Z.-Y. miRNA-1271 inhibits cell proliferation in neuroglioma by targeting fibronectin 1. *Mol. Med. Rep.* **16**, 143–150 (2017).

17. Daubon, T. *et al.* Deciphering the complex role of thrombospondin-1 in glioblastoma development. *Nat. Commun.* **10**, 1146 (2019).

18. Qi, C. *et al.* Thrombospondin-1 is a prognostic biomarker and is correlated with tumor immune microenvironment in glioblastoma. *Oncol. Lett.* **21**, 22 (2021).

19. DeSisto, J. *et al.* HGG-18. SINGLE CELL RNA-SEQ ANALYSIS OF PEDIATRIC HIGH-GRADE GLIOMA PATIENT SAMPLES IDENTIFIES COMMON GLIAL AND IMMUNE CELL TYPES THAT HELP TO EXPLAIN HETEROGENEITY AND TUMORIGENESIS. *Neuro. Oncol.* **25**, i43–i43 (2023).

20. Zahedi, S. *et al.* LGG-22. CHARACTERIZATION OF THE TUMOR MICROENVIRONMENT IN PEDIATRIC LOW-GRADE GLIOMA. *Neuro. Oncol.* **25**, i60–i61 (2023).

21. Wang, A. Z. *et al.* Single-cell profiling of human dura and meningioma reveals cellular meningeal landscape and insights into meningioma immune response. *Genome Med.* **14**, 49 (2022).

22. Brosseau, J.-P. *et al.* Human cutaneous neurofibroma matrisome revealed by single-cell RNA sequencing. *Acta Neuropathol Commun* **9**, 11 (2021).

23. Gonzalez, H. *et al.* Cellular architecture of human brain metastases. *Cell* **185**, 729-745.e20 (2022).

24. Yang, A. C. *et al.* A human brain vascular atlas reveals diverse mediators of Alzheimer’s risk. *Nature* **603**, 885–892 (2022).

25. Abdelfattah, N. *et al.* Single-cell analysis of human glioma and immune cells identifies S100A4 as an immunotherapy target. *Nat. Commun.* **13**, 767 (2022).

26. Zhao, Z. *et al.* Chinese Glioma Genome Atlas (CGGA): A Comprehensive Resource with Functional Genomic Data from Chinese Glioma Patients. *Genomics Proteomics Bioinformatics* **19**, 1–12 (2021).

27. Varn, F. S. *et al.* Glioma progression is shaped by genetic evolution and microenvironment interactions. *Cell* **185**, 2184-2199.e16 (2022).

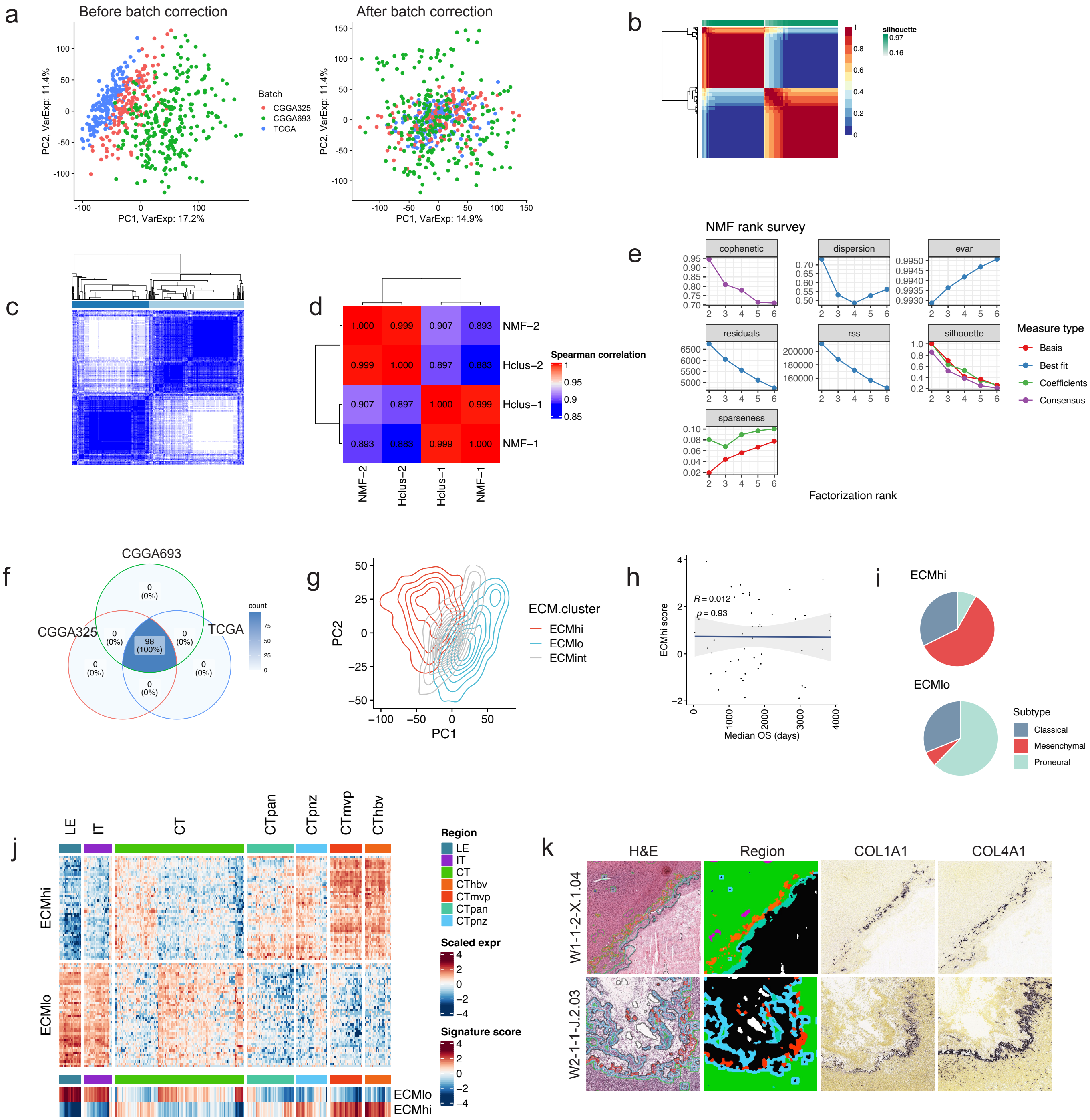

Supplementary Figure 1. Identification and characterization of ECM subtypes in brain tumors. **(a)** First and second principal components of batch-corrected (left) and uncorrected (right) datasets. **(b)** Consensus matrix obtained from 50 runs of non-negative matrix factorization. **(c)** Consensus matrix obtained from hierarchical clustering. **(d)** Spearman correlation between gene expression clusters obtained using NMF and hierarchical clustering. **(e)** Metrics used for estimation of NMF factorization rank. **(f)** Venn diagram showing overlap between of ECM$^{hi/lo}$ signature genes across three datasets. Each signature gene shows evidence of differential expression in all three datasets. **(g)** Principal component analysis of ECM^{hi}, ECM^{lo} and ECM^{int} brain tumor RNA-seq samples based on ECM gene expression. **(h)** Spearman correlation between median overall survival (OS) and average ECM^{hi} score in pediatric brain tumors. **(i)** Distribution of glioblastoma molecular subtypes in ECM^{hi} and ECM^{lo} tumors. **(j)** Signature gene expression in different anatomical regions of glioblastoma. **(k)** In situ hybridization and adjacent hematoxylin and eosin (H&E) sections annotated for different histologic regions of GBM tumors. ECM^{hi} hallmark genes *COL1A1*, *COL4A1* are expressed in CTmvp regions, suggesting that ECM^{hi} signature is spatially associated with GBM microvasculature. Labels on the left represent patient IDs. Anatomical regions are colored the same way as in **(j)**.

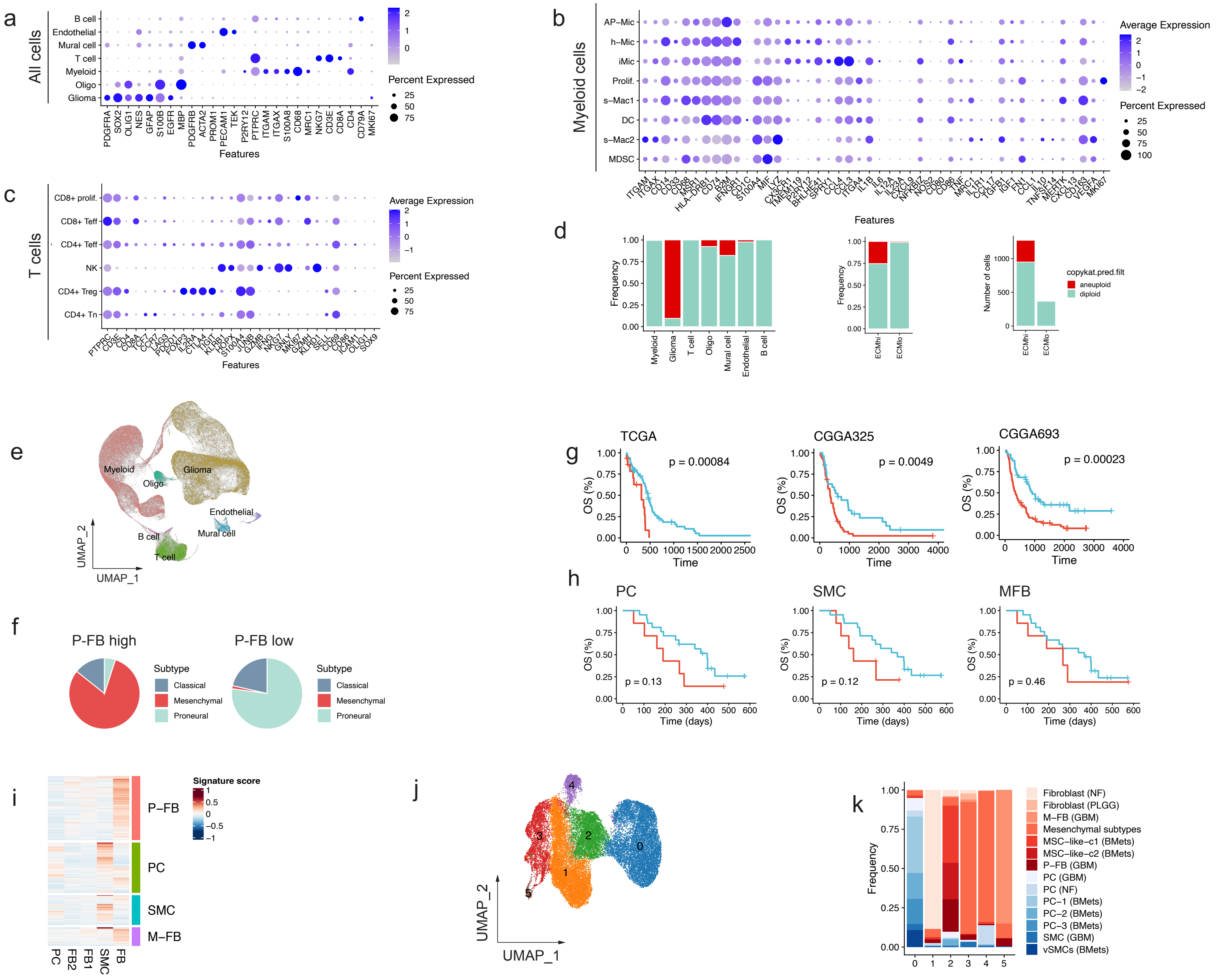

Supplementary Figure 2. Identification and characterization of perivascular fibroblasts in GBM scRNA-seq data. (a-c) Dot plots showing marker gene expression for different cell types. **(d)** Left: CopyKat predictions for all cell types; Right: CopyKat predictions for mural cells, grouped by ECM subtype, and shown both as frequency and absolute numbers. **(e)** UMAP embedding of GBM scRNA-seq data, colored by cell type. **(f)** Distribution of GBM molecular subtypes in tumors with high and low enrichment of the fibroblast-like signature. **(g)** Kaplan-Meier survival curves for ECM^{hi} (red) and ECM^{lo} (blue) GBM tumors. **(h)** Kaplan–Meier plot of overall and progression free survival for patients with high (red) or low (blue) presence of perivascular cell types (P values were calculated using log-rank test; PC – pericyte, SMC – smooth muscle cell, MFB – meningeal fibroblast). **(I)** Signature gene scores of murine perivascular cell types from Vanlandewijck et al. (2018). FB – fibroblast-like cell; SMC – smooth muscle cell; PC – pericyte. **(J)** UMAP embedding of batch-corrected stromal cell expression profiles from low-grade pediatric tumors, neurofibromas, meningiomas, and brain metastases, colored by cluster. **(K)** Barplot showing the per-cluster frequency of cell types from different tumors, related to **(I)**. Red denotes MSC and fibroblast populations, blue denotes PC and SMC populations.

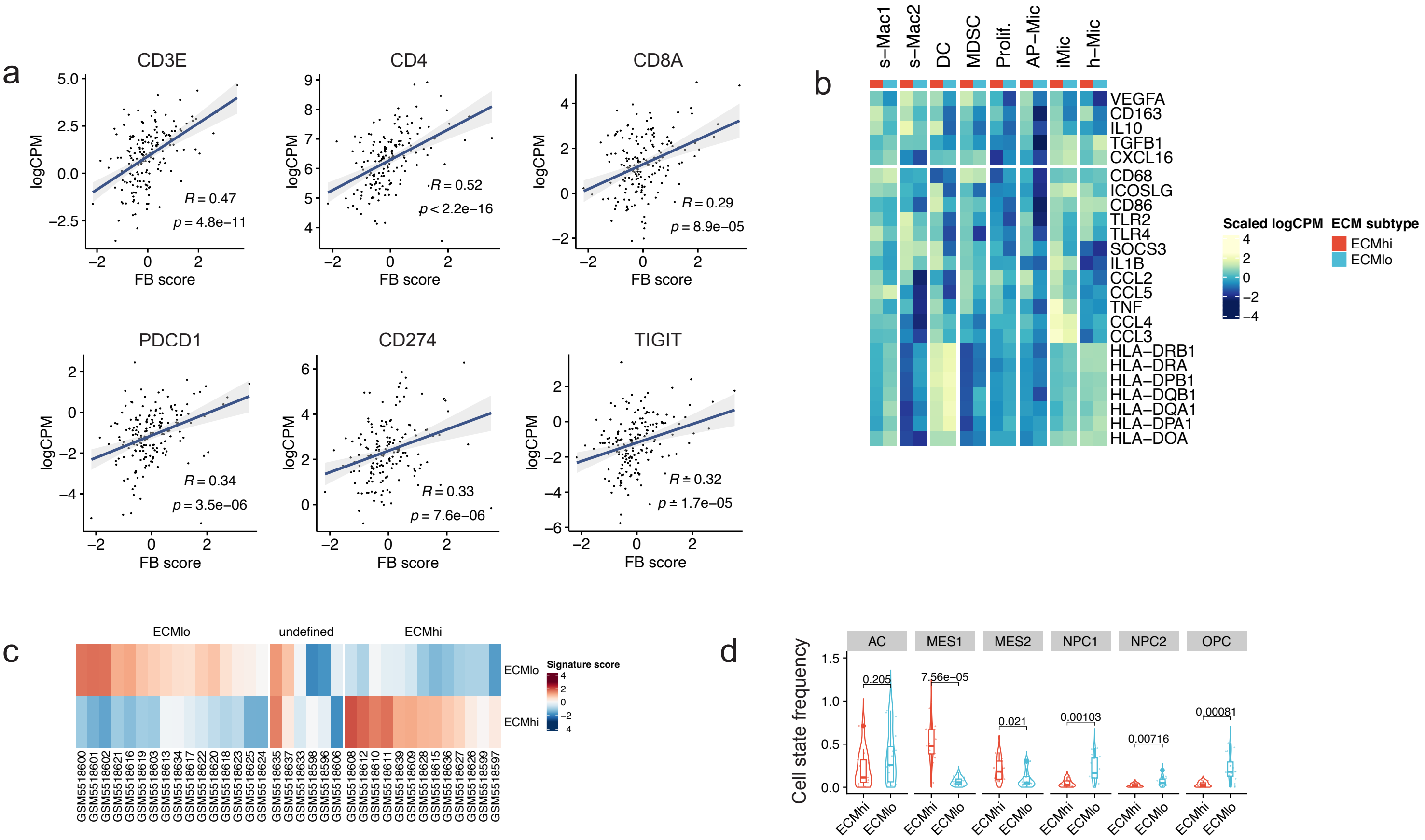

Supplementary Figure 3. Immune signatures associated with perivascular fibroblasts. (a) Spearman correlation between the perivascular signature score and immune-related genes. Spearman correlation coefficients together with P value are shown for each pair. **(b)** Pro- and anti-inflammatory gene expression in different myeloid subpopulations, grouped by ECM subtype. The line divides anti-inflammatory (top) and pro-inflammatory (bottom) genes. **(c)** ECM^{hi} and ECM^{lo} signature scores of pseudobulk gene expression profiles, grouped by ECM subtype. **(d)** Frequency of glioma cell states in ECM^{hi} and ECMlo tumors. OPC – oligodendrocyte progenitor cell-like; AC astrocyte-like; NPC – neural progenitor cell-like; MES – mesenchymal-like. Two-sided t-test. Benjamini-Hochberg adjusted P values are shown.
